# Supplementary material for: Identification of Freezing-Responsive microRNAs and Their Targets in Chinese Jujube by Small RNA and Degradome Sequencing
Source: Int J Mol Sci. 2026 Sep 4;27(17):7912. doi: 10.3390/ijms27177912 (PMC13566890; doi:10.3390/ijms27177912)
Supplement: Supplementary file 1 [file ijms-27-07912-s001.zip › Supplementary Material S2/Sequence and Genome ID of novel miRNAs.pdf]

Sequence and Genome ID of novel miRNAs

Number	miRNA	Mature Sequence	Genome ID
1	novel_miR_113	uuaccgauuccccccauuccc	NC_029689.1
2	novel_miR_205	uacuuguaaagaacucaaacgug	NC_029689.1
3	novel_miR_90	uaaugcuuacacaucuaauuccag	NC_029684.1
4	novel_miR_1	ucuuuccuaccccucccauacc	NW_015454295.1
5	novel_miR_120	uuagugauccuccggaagauc	NW_015453946.1
6	novel_miR_98	guaagacuaagcucaugagcuu	NC_029690.1
7	novel_miR_153	cuugcggaccgucuguaucauagc	NW_015453655.1
8	novel_miR_231	agauggauuuggagauggag	NC_029686.1
9	novel_miR_165	ccaguucauccaaacaccaug	NC_029683.1
10	novel_miR_122	uggagaagcagggcacgugc	NW_015454061.1
11	novel_miR_11	uggaucuuguuugacagcacu	NC_029682.1
12	novel_miR_86	uuuaaggacuaaagauugagg	NC_029689.1
13	novel_miR_216	uuacagguucgagaagaggca	NC_029688.1
14	novel_miR_89	ggauggucgaaggagagagaug	NW_015454851.1
15	novel_miR_111	ugucauguggcgugcuuauuuaau	NC_029686.1
16	novel_miR_80	auuucguuguauagaauggcgug	NC_029690.1
17	novel_miR_60	gcagcaucaccaagauuca	NC_029681.1
18	novel_miR_63	ucuuuccuaccccucccauacc	NW_015454792.1
19	novel_miR_13	uggaucuuguucgacagcacu	NC_029682.1
20	novel_miR_62	uuagauucacgcacaaacuc	NW_015453742.1
21	novel_miR_12	acuuccuucugcuaagcuaucacc	NC_029687.1
22	novel_miR_186	uuggcgugagagacuuggua	NW_015454295.1
23	novel_miR_48	aaguggaaugcuguucgaucgaauc	NC_029680.1
24	novel_miR_200	ucuuuccuauuccucccauucc	NW_015454792.1
25	novel_miR_59	cugccuauuccucccaugcc	NW_015454295.1
26	novel_miR_22	uuaucgacacuuuagaaaag	NW_015453658.1
27	novel_miR_8	cagggaagcggcagagcaugg	NC_029687.1
28	novel_miR_177	aucuuauaaaauacugcuuca	NC_029686.1
29	novel_miR_207	uuucaauugggucugaauu	NC_029681.1
30	novel_miR_118	ugcauuugcaccugcaccuuu	NC_029690.1
31	novel_miR_55	aguuguaguugcuugacgu	NW_015453522.1
32	novel_miR_147	uguguucucaggucgccccug	NC_029680.1
33	novel_miR_85	cuugcggauauccauaccugaua	NC_029684.1
34	novel_miR_106	aaauuggcugauauuaug	NC_029690.1
35	novel_miR_53	accgauccgugagcucagcuug	NC_029688.1
36	novel_miR_116	uuggcgugagagacuuggua	NC_029681.1
37	novel_miR_160	uugauugcagaaagaguuac	NC_029683.1
38	novel_miR_184	aaucuuucuguuucaccugaa	NC_029685.1
39	novel_miR_152	caugugcucuagcucuccagc	NC_029679.1
40	novel_miR_105	cgcuauccauccugaguuuc	NC_029689.1
41	novel_miR_188	uuccacagcuuucuugaacu	NC_029679.1

42	novel_miR_135	uggaucuuguuugacagcacu	NC_029682.1
43	novel_miR_9	ucacuaauacugcaguccucaugc	NC_029683.1
44	novel_miR_151	ucaggucauuggauggagagagu	NC_029680.1
45	novel_miR_161	uggaucuuguuugacagcacu	NC_029682.1
46	novel_miR_156	uuucuagaaucugaguauauuc	NC_029682.1
47	novel_miR_196	cgugauauugauucggcucauc	NW_015454528.1
48	novel_miR_164	uuacaagaguuggaauucaga	NC_029686.1
49	novel_miR_148	uuaucgacacuuuagaaaag	NW_015453537.1
50	novel_miR_154	uaaugcuuacacaucuaauuccag	NW_015453542.1
51	novel_miR_198	uggagaagcagggcacgugc	NW_015454179.1
52	novel_miR_7	acuugagacgaugaugauggcacau	NW_015453582.1
53	novel_miR_30	agauggauuuggagauggag	NC_029686.1
54	novel_miR_65	uuaaugaugccuccuuccuauagc	NC_029679.1
55	novel_miR_136	cgcacgcuagcggggaaccauc	NW_015453517.1
56	novel_miR_123	uggaucuuguucgacagcacu	NC_029682.1
57	novel_miR_142	cgugauauugauucggcucauc	NW_015454521.1
58	novel_miR_157	agagugauugucuugguucu	NC_029681.1
59	novel_miR_91	ggagaaguacaggcuggcuaugga	NC_029680.1
60	novel_miR_149	uugucugaauuuacaaucucg	NC_029685.1
61	novel_miR_26	aagacuugucuagaucaauagc	NW_015454027.1
62	novel_miR_104	accgauccgugagcucagcuug	NC_029688.1
63	novel_miR_190	caauuaauuuuuggauaugagc	NC_029688.1
64	novel_miR_221	uuuuuguuuuuuggggguug	NW_015453526.1
65	novel_miR_130	cgauuucugacacuuucucag	NC_029685.1
66	novel_miR_124	ucuggcuugucuaugaucugcgu	NC_029681.1
67	novel_miR_171	uuggcugauucucuauuuuuacag	NC_029686.1
68	novel_miR_191	caauuaauuuuuggauaugagc	NW_015454007.1
69	novel_miR_226	ggaaucuugaugaugcugcag	NC_029681.1
70	novel_miR_229	uuuuugguacaucacauucaugc	NW_015453447.1
71	novel_miR_31	uuaaguuugauugauagcu	NC_029687.1
72	novel_miR_92	uggaucuuguuugacagcacu	NC_029682.1
73	novel_miR_119	acagauauggucgagggg	NW_015453444.1
74	novel_miR_81	uuagugauccuccggaagauc	NW_015453946.1
75	novel_miR_72	caauuaauuuuuggauaugagc	NW_015453796.1
76	novel_miR_174	uuccacagcuuucuugaacu	NW_015453786.1
77	novel_miR_41	aaagaggaagagggguggug	NC_029682.1
78	novel_miR_195	aaaagauuugauggauuguuauga	NC_029690.1
79	novel_miR_220	gugccaugaacuugauccaga	NW_015453762.1
80	novel_miR_15	aaauucaauuugaacucuaccacc	NC_029681.1
81	novel_miR_134	ugauguauggaaugaagau	NC_029683.1
82	novel_miR_84	ucucugaucuucugcgacacggaa	NW_015453409.1
83	novel_miR_225	uugaauugagacucuaaguug	NC_029687.1
84	novel_miR_24	uaacucugauuucuguuauuugcag	NC_029679.1
85	novel_miR_77	ucuuuccuaccccucccauacc	NW_015454706.1

86	novel_miR_100	Uauuggauuucuuucuuug	NC_029682.1
87	novel_miR_139	uugccgacuccacccauuccca	NC_029681.1
88	novel_miR_182	cgugauauugauucggcucauc	NW_015458025.1
89	novel_miR_212	acuugagacgaugaugauggcacau	NC_029684.1
90	novel_miR_82	ucuuuccuauuccucccauucc	NW_015454295.1
91	novel_miR_32	acucucccucaaggcuucuag	NC_029686.1
92	novel_miR_18	uaagguguauaaacagaac	NC_029679.1
93	novel_miR_66	ucucugaucuucugcgacacggaa	NW_015457586.1
94	novel_miR_214	gguguggccugagaacauagg	NC_029688.1
95	novel_miR_145	cauggauuguuuguaugagu	NW_015453444.1
96	novel_miR_211	cgugauauugauucggcucauc	NW_015453608.1
97	novel_miR_2	uuggcugauucucuauuuuuacag	NC_029686.1
98	novel_miR_71	uuaucgacacuuuagaaaag	NW_015453428.1
99	novel_miR_88	uggagaagcagggcacgugc	NC_029680.1
100	novel_miR_115	ggauggucgaaggagagagaug	NW_015457068.1
101	novel_miR_47	ugguacggaucuguauguggac	NW_015453764.1
102	novel_miR_227	uaagguguauaaacagaac	NC_029680.1
103	novel_miR_19	uuggacugaagggagcuccuc	NC_029681.1
104	novel_miR_176	uguuaaugaucugacuuugaagg	NC_029680.1
105	novel_miR_125	uauauaacgauuguaaaugcuguc	NC_029680.1
106	novel_miR_54	uggaucuuguucgacagcacu	NC_029682.1
107	novel_miR_146	ugccaaaggagaguugcccug	NC_029681.1
108	novel_miR_162	uuacagguucgagaagaggca	NC_029684.1
109	novel_miR_107	gagagaauuguaaggguuuugaga	NW_015453660.1
110	novel_miR_17	uaagguguauaaacagaac	NC_029679.1
111	novel_miR_155	ccugagauuuuguaagagcca	NC_029682.1
112	novel_miR_49	ggauggucgaaggagagagaug	NW_015455128.1
113	novel_miR_192	augggcuaauagcaucuccaaugg	NC_029679.1
114	novel_miR_75	acggugaagaaaaauguuuuu	NC_029682.1
115	novel_miR_167	uggaucuuguucgauggcacuc	NC_029682.1
116	novel_miR_222	aguagagacaugguaguucaaa	NC_029682.1
117	novel_miR_178	uuaucgacacuuuagaaaag	NW_015453403.1
118	novel_miR_79	uuagauucacgcacaaacucg	NC_029683.1
119	novel_miR_166	uggaucuuguucgacagcacu	NC_029682.1
120	novel_miR_213	ccgauugagcugcucuuuugacuc	NW_015454285.1
121	novel_miR_16	uacuuguaaagaacucaaacgug	NC_029683.1
122	novel_miR_204	uggacuuugcugaauacuaca	NW_015453527.1
123	novel_miR_68	uagaauuccauaacaauccauc	NW_015454086.1
124	novel_miR_50	uggaucuuguucgauggcacuc	NC_029682.1
125	novel_miR_3	gaguaguucuagaucuggucc	NW_015453449.1
126	novel_miR_189	cuuucuuucucuuccucu	NC_029690.1
127	novel_miR_170	cgugauauugauucggcucauc	NW_015454185.1
128	novel_miR_103	gacgaacuguauuuaugaauaugc	NC_029684.1
129	novel_miR_129	cgaagcccggaggaagagugg	NC_029689.1

130	novel_miR_64	ccgggaugauggaagaugcugagg	NC_029686.1
131	novel_miR_33	uugauuguuuucauucugaua	NC_029682.1
132	novel_miR_185	uggaucuuguucgauggcacuc	NC_029682.1
133	novel_miR_181	uuccaucucuugcacacug	NC_029686.1
134	novel_miR_133	cuucaagaucucugaguuugg	NC_029686.1
135	novel_miR_143	gaaaaagaaccguuagaca	NC_029689.1
136	novel_miR_126	uacuugaugauguauggaaug	NC_029683.1
137	novel_miR_159	ugaucuguuuaacucgaaauugac	NW_015453459.1
138	novel_miR_34	uggaucuuguuugacagcacu	NC_029682.1
139	novel_miR_44	uggaucuuguucgacagcacu	NC_029682.1
140	novel_miR_179	uuuguugggaagaagauagcag	NC_029682.1
141	novel_miR_29	uuuacugggcuugggcuu	NW_015453467.1
142	novel_miR_93	ucucugaucuucugcgacacggaa	NW_015454151.1
143	novel_miR_202	ucaggucauuggauggagagagu	NC_029680.1
144	novel_miR_36	guguuaguugucauuuuagugcu	NC_029685.1
145	novel_miR_138	caggacuagaagguugggca	NC_029689.1
146	novel_miR_51	uuggcgugagagacuuggua	NW_015455326.1
147	novel_miR_158	aauaggccaugaaugagga	NC_029684.1
148	novel_miR_37	ucaggucauuggauggagagagu	NC_029680.1
149	novel_miR_97	gcuaaauggaauagacaca	NC_029681.1
150	novel_miR_117	uuagugauccuccggaagauc	NW_015453946.1
151	novel_miR_67	cuggauccuuagcucacuuuaag	NW_015453610.1
152	novel_miR_46	aagcucaggagggauagcgcc	NC_029680.1
153	novel_miR_58	ggauggucgaaggagagagaug	NW_015455534.1
154	novel_miR_183	cagaauuucuaaacaucaaaucg	NW_015453474.1
155	novel_miR_168	auagacggacuguauauauauau	NW_015453691.1
156	novel_miR_87	caaauaaugaucuacguaua	NW_015453496.1
157	novel_miR_114	uggaucuuguuugacagcacu	NC_029682.1
158	novel_miR_57	cgugauauugauucggcucauc	NW_015454278.1
159	novel_miR_95	uuugugucuuuccuuugua	NC_029682.1
160	novel_miR_40	caaaugaguguggauacccagga	NC_029683.1
161	novel_miR_208	uuggcgugagagacuuggua	NW_015454706.1
162	novel_miR_96	uauuuaacaacucuuugaauguag	NC_029685.1
163	novel_miR_25	ucgcucuguucccucugaagauu	NC_029687.1
164	novel_miR_132	uuggcgugagagacuuggua	NC_029681.1
165	novel_miR_52	uuccacagcuuucuugaacu	NC_029679.1
166	novel_miR_173	aaagaauuugauggauuguuauga	NC_029683.1
167	novel_miR_101	ucuuuccuaccccucccauacc	NC_029681.1
168	novel_miR_210	ugguucuugaacaucaugcuga	NC_029680.1
169	novel_miR_193	ucacuaauacugcaguucucaugc	NC_029683.1
170	novel_miR_99	ucuuuuauggcuguggauuugcu	NC_029679.1
171	novel_miR_128	caugcgaacuguaucaaaaac	NC_029679.1
172	novel_miR_56	gguaguuugaucgugaaauu	NW_015453479.1
173	novel_miR_172	aagacuugucuagaucaauagc	NW_015455874.1

174	novel_miR_144	cucuaucacugacauacguau	NC_029684.1
175	novel_miR_110	uauuuugugcugauuucaug	NC_029680.1
176	novel_miR_14	uuccacagcuuucuugaacu	NC_029679.1
177	novel_miR_27	uggagaagcagggcacgugc	NC_029687.1
178	novel_miR_74	uuccacagcuuucuugaacu	NC_029679.1
179	novel_miR_197	uggcugaauagaugaauu	NC_029688.1
180	novel_miR_215	uaauugugagaaugugugua	NC_029684.1
181	novel_miR_194	cuucaugcggacuacaguauuggu	NC_029688.1
182	novel_miR_43	aggaguauggaaaauugugc	NC_029684.1
183	novel_miR_187	agucaaauugaauggaugauug	NC_029681.1
184	novel_miR_150	aucaacuuugguguaguacgca	NW_015454225.1
185	novel_miR_206	cgugauauugauucggcucauc	NW_015454460.1
186	novel_miR_61	uauauaacgauuguaaaugcuguc	NC_029680.1
187	novel_miR_163	uugucugaauuuacaaucuca	NC_029685.1
188	novel_miR_228	gcggcaucaucaggauucaca	NC_029680.1
189	novel_miR_73	acgaugugugu aucuagggaa	NW_015453508.1
190	novel_miR_175	cuugcggauauccauaccugaua	NC_029679.1
191	novel_miR_39	aagacuugucuagaucaauagc	NW_015454071.1
192	novel_miR_131	cgugauauugauucggcucauc	NW_015454519.1
193	novel_miR_4	ugaugugcucguggaacugaacc	NC_029689.1
194	novel_miR_223	uccguauuguagaauuauccuaua	NC_029679.1
195	novel_miR_209	ggauggucgaaggagagagaug	NW_015454862.1
196	novel_miR_217	uaucugcaucuguauagcuuuc	NC_029679.1
197	novel_miR_38	gugugugucuugacuguugagagc	NC_029689.1
198	novel_miR_10	gugugugucuugacuguugagagc	NC_029690.1
199	novel_miR_70	uucuguaguuccuggaaucug	NC_029686.1
200	novel_miR_69	caucaucugugcucucuaucu	NC_029683.1
201	novel_miR_137	uuaucgacacuuuagaaaag	NW_015453428.1
202	novel_miR_199	uaagguguauaaacagaac	NW_015453403.1
203	novel_miR_21	uuccacagcuuucuugaacu	NW_015453786.1
204	novel_miR_108	ucuuuccuauuccucccauucc	NC_029681.1
205	novel_miR_6	ucuuuccuauuccucccauucc	NW_015454706.1
206	novel_miR_42	uggaucuuguuugacagcacu	NW_015453762.1
207	novel_miR_230	gguaguuugaucgugaaauu	NC_029680.1
208	novel_miR_76	ucacuaauacugcgguucucaugc	NC_029689.1
209	novel_miR_219	ucuuuccuaccccucccauacc	NC_029681.1
210	novel_miR_83	ucauauuuguuccaucgccugugg	NC_029680.1
211	novel_miR_180	cgcugccaaagaugucgccaug	NW_015454364.1
212	novel_miR_201	augacuguucaaaauuuca	NC_029680.1
213	novel_miR_112	aagguacucuguaaaaacg	NC_029685.1
214	novel_miR_78	ugcuaacuuguagacguugugagg	NC_029682.1
215	novel_miR_5	aaauucuauggugaggacggu	NC_029681.1
216	novel_miR_140	guaagacuaagcucaugagcuu	NC_029690.1
217	novel_miR_169	uuagugauccuccggaagauc	NC_029685.1

218	novel_miR_35	aauagaggauaaguaaacag	NC_029685.1
219	novel_miR_121	uuagauucacgcacaaacuc	NW_015453399.1
